# Effects of Automotive Test Parameters on Dry Friction Fiber-Reinforced Clutch Facing Surface Microgeometry and Wear

**DOI:** 10.3390/polym13223896

**Published:** 2021-11-11

**Authors:** Roland Biczó, Gábor Kalácska, Tamás Mankovits

**Affiliations:** 1 Institute of Technology, MATE, Páter Károly u. 1, H-2100 Gödöllő, Hungary; kalacska.gabor@uni-mate.hu; 2 Department of Mechanical Engineering, Faculty of Engineering, University of Debrecen, Ótemető u. 2-4, H-4028 Debrecen, Hungary; tamas.mankovits@eng.unideb.hu

**Keywords:** dry friction, hybrid composite, surface roughness, wear, activation energy, clutch

## Abstract

Wear and surface microgeometry aspects of fiber-reinforced hybrid composite dry friction clutch facings are revealed in a novel way: after different, real life automotive tests during their lifetime. This study examines and reveals the tribological response of friction material surfaces to real life application conditions with two different facing diameters and in two directions. Along the increasing activation energy scale, wear values increased according to two different trends, sorting tests into two main groups, namely ‘clutch killer’ and ‘moderate’. Wear results also highlighted the influence of mileage and test conditions, with clutch killer tests also creating considerable wear-more than 0.1 mm-at inner diameters: 1% higher wear was generated by 90% higher mileage; another 1% increment could be caused by insufficient cooling time or test bench-specific conditions. Surface roughness values trends varied accordingly with exceptions revealing effects of facing size, friction diameter and directions and test conditions: small (S) facings produced significantly decreased Rmax roughness, while large (L) and medium (M) size facings had increased roughness values; Rmax results showed the highest deviations among roughness values in radial direction; tests run with trailer and among city conditions resulted in more than 2% thickness loss and a 40–50% roughness decrease.

## 1. Introduction

In automotive applications, the purpose of utilizing a clutch is to transfer kinetic energy in the form of modulated torque of a rotating crankshaft coupled to a power source towards a transmission system and further to the wheels of the vehicle. [1] The commonly used single disc clutch system consists of a flywheel, a clutch disc with composite friction facings and the clutch itself with the releasing diaphragm spring. Following a remarkable friction material development throughout the past century, fiber-reinforced hybrid composites became the most used dry friction materials in such applications [2].

Modern hybrid composites consist of a great variety of components, which makes their identification and characterization rather complex.

These components of a hybrid composite material are usually sorted into groups based on their mechanical role and function. However, one single component usually affects many different characteristics besides the targeted aspect: even reinforcing fibers play a critical role regarding tribological–friction and wear–properties [3]. Furthermore, even binders can modify friction behavior of composites, as was found by Wongpayakyotin et al. [4], while Bijwe [5] stated that aromatic polyamide guarantees good wear properties and a stable coefficient of friction. Furthermore, copper turned out to be a component that improves all major properties of frictional materials in the investigations by Kumar et al. [6].

On the other hand, filler materials with various volume percentages were found by Basavarajappa et al. [7] to make a significant contribution to dry sliding characteristics of glass-epoxy composite. Furthermore, Chang et al. [8] investigated the tribological properties of two kinds of high temperature-resistant thermoplastic composites with short carbon fibers (SCF), graphite flakes, and sub-micro particles via a pin-on-disc setup. They concluded that base polymer wear resistance and load-carrying capacity can be effectively enhanced by conventional fillers while frictional coefficient and wear rate can be further reduced at elevated temperatures through the addition of those particles. While Olifirov et al. [9] examined the coefficient of friction and wear resistance among other properties of fluorinated ethylene propylene filled with Al–Cu–Cr quasicrystals, polytetrafluoroethylene, synthetic graphite, and carbon black, finding that a certain proportion of fillers leads to optimized tribological properties. Rangaswamy et al. [10] reported a comparison of wear resistance of hybrid, carbon nanotube-filled and neat E-glass, Kevlar, epoxy composites, finding the former resulted in less wear. Bajpal et al. [11] on the other hand proposed utilization of different fillers for cyanate ester thermoset resin to widen its industrial applicability by improvements in tribo-mechanical properties. Graphite was found to be a good candidate, offering a lower specific wear rate and lower friction value.

Even agriculture fiber wastes such as corn, sugar bars and palms fibers were found to be capable of increasing the friction coefficient while decreasing wear in an investigation by Bakry et al. [12], with the goal of replacing composite components with environmentally friendly friction materials for brake linings and clutch facings. Tamayo et al. [13] managed to produce better behavior of brake pads regarding the coefficient of friction by adding recycled end-of-life tire rubber particles to the composite brake materials. Better adhesion enhanced the long-life behavior. Jubsilp et al. [14] found that ultrafine full-vulcanized acrylonitrile butadiene rubber particles modify the behavior of asbestos-free polybenzoxazine friction composites so that the friction coefficient and wear resistance is enhanced optimally, and the composite becomes acceptable as a replacement material for asbestos-based friction brake pads. Therefore, the effect of components on the performance is so complex, that to achieve the optimal composition is rather art than science [15]. Since there are many investigation methods in this subtopic, the tribological effects of the components are well-known. Their synergy makes it difficult to obtain the optimal composition.

Surface roughness is a governing parameter in tribological systems. Kubiak et al. [16] found that initial surface roughness significantly influences the coefficient of friction at the transition between partial slip and full sliding. Furthermore, Fernandes et al. [17] investigated effects of cast discs with modified surface roughness values on the wear rate and friction coefficient (COF) of a clutch system. They found that a smooth surfaced cast iron disc reduced wear rate and contributed to a higher and more stable running-in period friction coefficient level by not damaging the developed tribofilms and maintained them more stably due to a reduction in contact area stresses at the highest tribometer test. Modifying *pv* (surface pressure multiplied by velocity) values towards severe conditions affected running-in COF fluctuation. Besides these approaches, Abdullah et al. [18] examined the effects of surface roughness on thermoelastic behavior through generated heat and created a novel axisymmetric finite element model to study the sliding period of the clutch with actual surface roughness considered instead of common flat surface models. They concluded that the magnitude and the distribution of the contact pressure is highly affected by roughness of contact pairs.

Friction coefficient and wear rate are the essential components to describe the tribological behavior of frictional hybrid composites. Their examination is a complex scientific field that involves the tests related to these values, or the fading effect and the third body phenomena. Pin-on-disc frictional models are helpful to observe a transition from mild to severe wear under specific load conditions governed by hardness, normal load, surface roughness, sliding velocity and temperature through time [19]. On the other hand, it is a common problem that test devices only allow investigating the starting and final conditions with the amount of friction debris. The mechanism of the transition can only be approximated. Fernandes et al. [20] found that the stability of multi-layer friction films can be influenced by sliding conditions and that removing wear debris results in increasing friction level and reducing wear rates. Fidlin et al. [21] on the other hand concentrated on the contact influencing phase transition from solid organic material to gas in dry clutches. Phase transition resulting in decreasing transmissible torque is called the fading effect. Qualitative explanations were given of both fading and recovery effects overcoming numeric problems via asymptotic expansion for the pressure field in a newly developed model for the dynamical behavior consequences of outgassing. They found permeability of the contact layer to be the main effective parameter.

Investigations often rely on models besides empirical data. The commonly utilized frictional models can be categorized into two groups: empirical models and models based on physical equations. The former work better with certain applications preferring the usage of velocity dependency; the latter can describe friction before slip [22].

Among others, Duque et al. [23] evaluated the dynamic Stribeck friction model beside the Coulomb model to compare their applicability in dry clutch engagement energy simulations during vehicle launch. Their conclusion was that the Coulomb model–recreating molecular effects and capable of surface adhesion consideration–showed a more acceptable result. On the other hand, Chu et al. [24] used the recursive least square method with forgetting factor approach in order to identify the friction coefficient of a dry clutch.

Grzelczyk and Awrejcewicz [25] carried out mathematical and numerical analysis of a dry clutch contact supported by experiments. They took wear properties and flexibility of friction materials into consideration in their investigations with differential and integral wear models. Including elasticity of the friction materials made possible more thorough consideration of the wear processes, and calculation of wear distributions on the entire contact surface along with transmitted torque. Li et al. [26], on the other hand, presented a method to predict wear of a paper-based, wet clutch friction lining subjected to repeated engagement cycles with the fact in mind that wear behavior of the friction material is closely related to both the thermal degradation of organic fibers and the stress condition at the sliding interface. Predictions from their model were compared to the two-stage wear rate phenomenon, revealing that the model is capable of considering this trend.

To conclude, stability of the fiction coefficient and wear resistance of polymer composite friction materials for clutches of the present day are determined by requirements derived from the loads and environment, in the same way as strength against transient mechanical impacts and high rotational velocity, deformability and heat resistance against thermal loads [2]. Therefore, tribological scholars often detail the effects of components and manufacturing parameters on frictional properties of the material.

For instance, the friction coefficient and wear are usually observed and evaluated via pin-on-disc test setups. Furthermore, tribological behavior along with harmful effects of the frictional contact is often described in empirical or physical equation-based models. The reasons for harmful effects of the frictional contact, the imbedding phenomena, the fading effect, and the judder, are mainly clarified in the literature. Modifying the friction material does not always prevent these phenomena; contact parts also affect fueling contact model creation attempts.

The coefficient of friction is one of the main governing parameters in these contact models. This parameter not only varies during one clutch engagement due to temperature, etc., but also during lifetime of the clutch as effects of wear and applied energy are getting more and more significant. On the other hand, today’s development trends require numerical models for quick and effective optimization (with, for example, testing cost reduction in mind), but in spite of considerable research efforts to develop materials by modifying one or two components before validation through experiments, there is no general complex material model to describe hybrid friction composite facing material behavior. The chemical reactions, physical changes and effects of surface discontinuities are not yet clear, making the safety factor during development of friction systems non-optimal.

Moreover, despite the many investigations carried out regarding the friction behavior of dry sliding hybrid composite clutch facings, there are not many approaches that examine samples from facings after real life or test track usage in actual vehicles, and comparison of tribological performance of the same material with different preliminary activation energy levels on the friction surface and its effects on the stability of the coefficient of friction.

The aim of this paper was to reveal the tribological, lifetime affected aspects of dry friction hybrid composite clutch facings, concentrating on wear and surface roughness values, possible correlations between them and effects on each other. Results are awaited to support finite element models for clutch development since responses of the tribological system of a dry clutch should not only be a function of contact temperature, but also of more comprehensive effectsfor example. applied energy–considered during its lifetime.

## 2. Applied Composite Friction Material, Its Identification and Tribo-Thermomechanical Integration

The friction material investigated in this study was a conventional dry friction hybrid composite facing material manufactured by the scatter wound process. Identification and mechanical and thermal characterizations for this material were carried out in our recent study [2]. To understand our current aim–to integrate tribological aspects, such as effects of varying load cases on wear and surface roughness into a thermomechanical model–a short summary is required to sum up the thermomechanical identification results.

**Figure 1:**
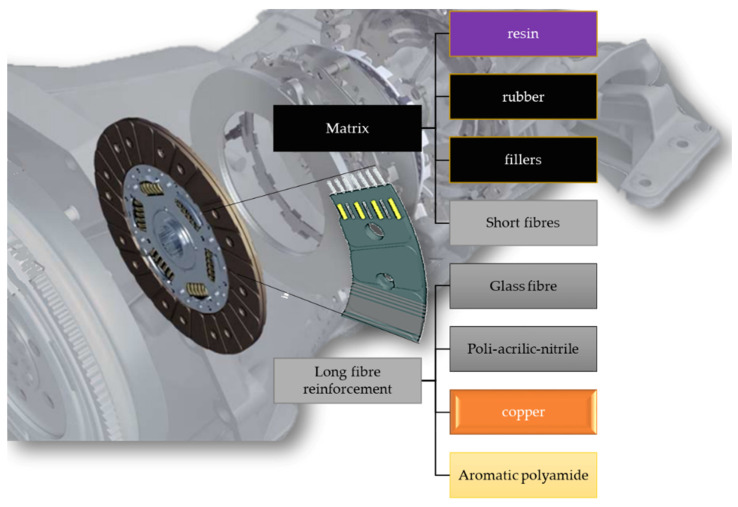
Structure and components of the hybrid composite friction facing.

In this friction material, the long fiber reinforcement, the so-called yarn, consisted of glass fiber, aromatic polyamide, copper, and poly-acrylic-nitrile (PAN), while the matrix is an industrial secret, hence the required identification. As a first step of this approach, infrared spectral analysis revealed that the matrix of the investigated hybrid composite that coated the yarn was not polyester-based, but an epoxy-based resin: a short fiber-reinforced composite itself, including a melamine-modified epoxy phenol resin filled with compounded rubber and other components such as sulfur, aromatic polyamide as short fiber reinforcement, and other filler materials as illustrated in Figure 1.

Test samples for characterization investigations were created via abrasive water jet machining, which turned out to be the most effective method for such a purpose regarding fiber-reinforced composite materials. Via tensile test, Iosipescu shear test, two directional strain measurement, Hyperion thermal expansion test, differential scanning calorimetry and measurements on Lee’s apparatus, mechanical and thermal properties were determined respectively. Mechanical properties were determined separately for the reinforcement and the matrix. As Table 1 summarizes, it was revealed that the matrix component group can be considered with a Young’s modulus of 4290 MPa, Poisson’s ratio of 0.38 and shear modulus of 1290 MPa, while the fiber-reinforcement component group was characterized by a Young’s modulus of 27,300 MPa, Poisson’s ratio of 0.2 and; shear modulus of 11,380 MPa. Values of component groups were united via ruling mixtures into a mechanical stiffness matrix for the whole material, while the coefficient of thermal expansion between 0 °C and 180 °C, the specific heat versus temperature between 50 °C and 240 °C, and the thermal conductivity coefficient as 0.398 W/(m·K) were defined by thermal investigations in three geometrically specified directions.

**Figure 2:**
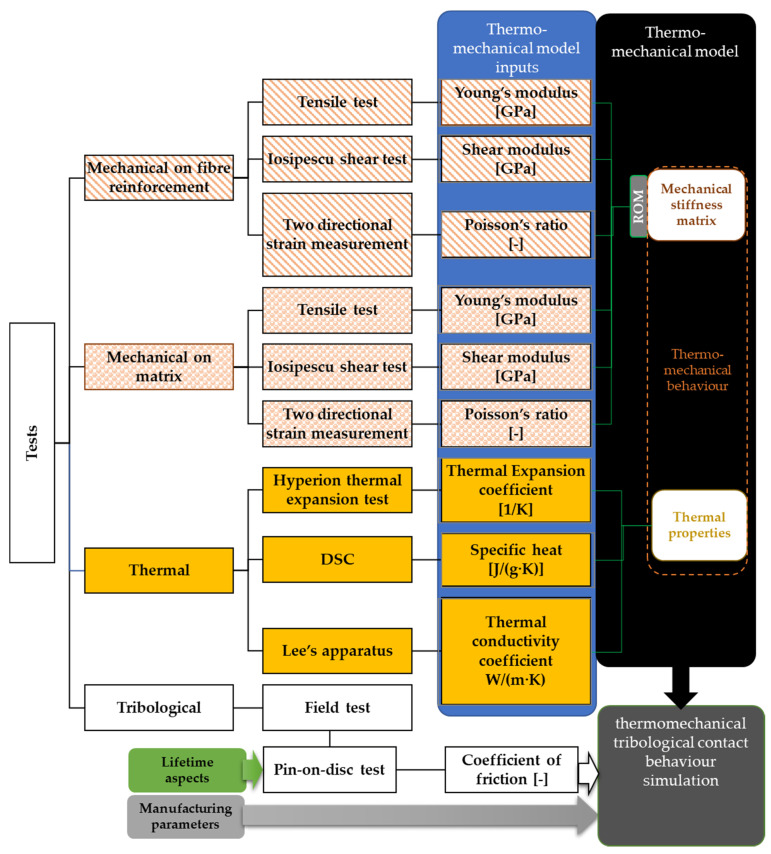
Our developed thermomechanical characterization and modeling method with tribological aspects.

The identification and characterization approach itself can be a reference and a novel guidance for material identification and thermomechanical characterization methods from almost raw material properties for similar complex composite materials. As illustrated in Figure 2, the results provide input parameters for thermomechanical simulation contact model development covering tribological aspects of dry clutches, which is the aim of current investigations.

## 3. Tribological Aspects: Wear Conditions and Surface Microgeometry

Kostetsky considered tribosystems to be open thermodynamic systems capable of exchanging energy and matter with the environment. Two fundamental phenomena were noted to be responsible for processes occurring in case of friction: activation (when free energy of materials in the tribosystems increases) and passivation (when it decreases). Tribological processes are characterized by the ratio and/or balance of activation and passivation energy [27].

During a dry friction automotive clutch engagement, assuming constant friction, the *pv* value is proportionate to the dissipated thermal energy that in the form of surface activation energy transforms the surface microgeometry and leads to wear phenomena besides temperature rise and interface material transformation-associated entropy changes [28]. The friction power intensity concept was introduced by Matveesky [29] in the form of energy dissipated per unit of contact area back in the 1960s. Not so long ago interfacial shear work in frictional contacts was considered by Fouvry et al. [30] to quantify wear rates and lifetime of coatings with transposed local wear energy analysis. The created novel characteristic of the system, namely the wear energy coefficient, led to rationalized wear quantification so classification of studied tribosystems became possible. Ramalho and Miranda introduced a new, energetic approach to analyze the wear data so that tribological material response could be characterized and prediction of working life could be carried out [28].

The aim of our investigation was to compare surface microgeometry characteristics, thickness loss of facings (wear, resistance of the friction surface, etc.) and tribological performance of dry friction clutch facing surfaces that had been used in automotive tests with different surface activation energy values. The goal was to be able to predict facing material tribological performance and behavior during a clutch’s lifetime.

In the automotive industry, to guarantee that the clutch disc and its friction material meet their requirements, a handful of tests are available. These tests were conducted according to automotive industry development and inspection routines among varying conditions following best practice (see Table 2). One of these tests was run on a test bench that simulated, among laboratory conditions, such tribological circumstances that the clutch experiences in a passenger car driven on a highway: long runs after maximal acceleration with longer down times to make investigation of effects of cool-down periods possible. During 60–70% of the total testing time maximum acceleration conditions were recreated; 30–40% of total time simulated countryside driving, while in 1–2% of the total testing time the clutch met idle conditions for cooling. The test bench was built so that the clutch engaged with a flywheel that had a moment of inertia to meet the applied energy values during real life use on prescribed road types. Other tests were carried out using test vehicles. During two of these, average customers were driving the cars with or without a trailer throughout a whole year on the streets, reaching high mileage values with frequent shifting. Another one was run on a test track with professional drivers intentionally accelerating under full load with a high number of shifts to create thermal peaks. Two other tests were to recreate hill start conditions with or without a trailer, repeatedly actuating the clutch to reach high temperatures such as 300–400 °C while the vehicle started to move upwards on various angled slopes. Table 1 contains a detailed description of one of these test benches and five of these vehicle tests for ensuring dry clutch facing quality.

After these preliminary tests carried out with single disc manual transmission clutches with dry friction fiber-reinforced friction facings, samples were cut from friction surfaces on the flywheel side to be examined for tribological aspects. This way we had varied initial remaining wear conditions for our specimens, recreating tribological conditions as a function of varying lifetime parameters. Abbreviations used for test names are: T: test bench, H: highway, V: vehicle, C: city, VT: vehicle + trailer, R: testing ring (test track), RS: testing ring + hill start).

Determining surface activation energy for these tests provides an opportunity to compare and highlight their intensity and tribological effects. During the time (t) the clutch pedal is released, the dissipated energy for a single engagement indicated by temperature rise is a function of transmitted clutch torque (T_c_, [Nm]) and rotational velocity difference [ω_r_, [rad/s]) between coupling surfaces, as stated in Equation (1) [31]:

(1) $$E=\int_{0}^{t}T_{c}\cdot \omega_{r}\left(t\right)\mathrm{dt},$$

where according to Equation (2) the transmitted torque is function of the number of friction surfaces (z), the friction coefficient (µ), the friction radius (R_m_) and the clamp load (F_m_):

(2) $$T_{C}=z\cdot \mu \cdot F_{m}\cdot R_{m}.$$

Thermal load of the clutch can be calculated according to Equation (3) [32]:

(3) $$W=\frac{1}{2}\cdot m\cdot {\left(\frac{r_{\mathrm{dyn}}}{i_{1\mathrm{stgear}}\cdot i_{\mathrm{diff}}}\right)}^{2}\cdot {\left(2\cdot \pi \cdot n_{\mathrm{eng}}\right)}^{2}\cdot \frac{1}{1-\frac{T_{\mathrm{drag}}}{T_{\mathrm{engmax}}^{}\cdot \chi }},$$

where r_dyn_ is the dynamic rolling radius, i_1stgear_ is the first gear ratio, i_diff_ is the differential ratio, n_eng_ is the engine speed, T_engmax_ is the maximum engine torque, χ is an engine type-dependent torque factor and the drag torque T_drag_ is function of the vehicle mass.

For a certain diesel engine passenger car with a total weight of ~1300 kg and manual transmission with a 1st gear transmission ratio of 4.23 and differential ratio of 3.46, operating a clutch with facing outer/inner diameters of Ø228/Ø160 mm, the energy values for actuation in different circumstances and as building blocks of prescribed automotive tests are as Table 3 illustrates. These energy values are proportional to the *pv* value describing the different clutch engagement profiles [28].

However, during the tests described above many clutch engagements occurred and many tests were run in repeated cycles with different blocks from those in Table 2. Therefore, other fundamental quantitative parameters were required to be able to categorize the different usage profiles. One of these fundamental parameters is the number of shifts applied during a test per kilometer. This value equals the number of times the engagement happens between the flywheel and the pressure plate. During tests run on highways, this number was 0.7 per km, country side usage provided 5–7 shifts per kilometer, while in a city this value was the highest: 18–27. However, during tests including hill starts more than a hundred actuations happened.

Recreating the preliminary tests from these actuation profiles shown in Table 3, and multiplying the average number of shifts per mile with average applied energy per shift (Table 3) per friction area, the test intensity (average applied energy along 1 km mileage on 1 cm^2^ of the friction surface) of each investigation could be calculated and a hypothetical comparative scale could be created, as Table 4 illustrates.

Based on this, the intensity scale of the preliminary tests is as follows: **TH–VRS–VRTS–VC–VTC–VR**.

Taking mileage into consideration, the total energy can be calculated. Different sized dry friction fiber-reinforced clutch facings–from which our laboratory pin-on-disc test samples were cut–had been preliminarily loaded according to the matrix illustrated in Table 5. This matrix is actually a coordinate system characterized by the determined preliminary test intensity and mileage axes. Specimens were coded according to their load case position in this matrix as follows: TEST-mileage-size (based on diameters), where mileage values correspond to 1000 km (double 0 beginning highlighting 0.0x), while size values were S: Ø228/Ø160; M: Ø240/Ø160; L: Ø240/Ø155 as outer and inner diameter values. Within the same test category, larger diameter clutch facings were used for slightly higher clamp load applications, hence the diameter order along intensity.

Mileage is a common parameter to mark the lifetime of automotive components. Multiplying it with test intensity and activation surface values, the total surface activation energy could be calculated for all of our samples.

To examine or prove the test intensity order hypothesis, with which a tribological load consequence and prediction map was to be set up for dry friction single disc clutches, first the wear values of the clutch discs after the tests were examined. Table 6 illustrates results of thickness loss measurement on facings from different tests. Wear was examined along the inner (di) and the so-called working (dw)–initial working–diameter of the facings. Deviation is the calculated sigma (σ) based on 10 repeated measurements.

Table A1 details the same wear results, but compared to the initial nominal thickness values of the friction facings presented in Table 5.

**Figure 3:**
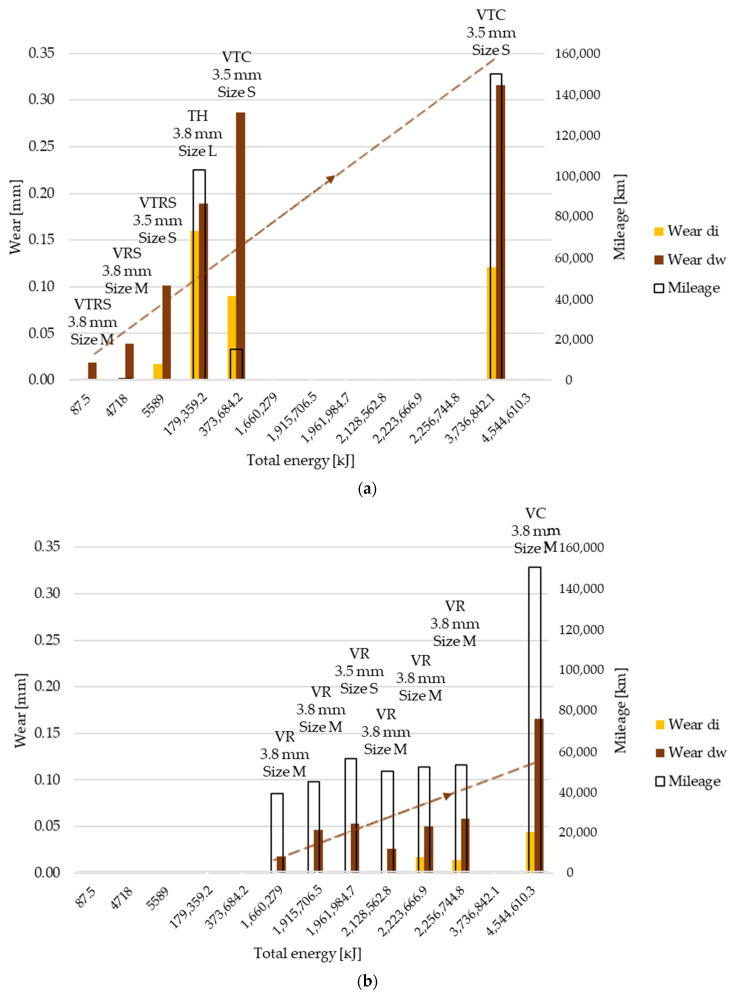
(**a**) Wear values on inner and working diameter with mileage along Joule-scale–trend 1: increasing, clutch killer load cases: VTRS, VRS, TH, VTC. (**b**) Wear values on inner and working diameter with mileage along Joule-scale–trend 2: increasing, moderate load cases: VR, VC (high mileage).

**Figure A1:**
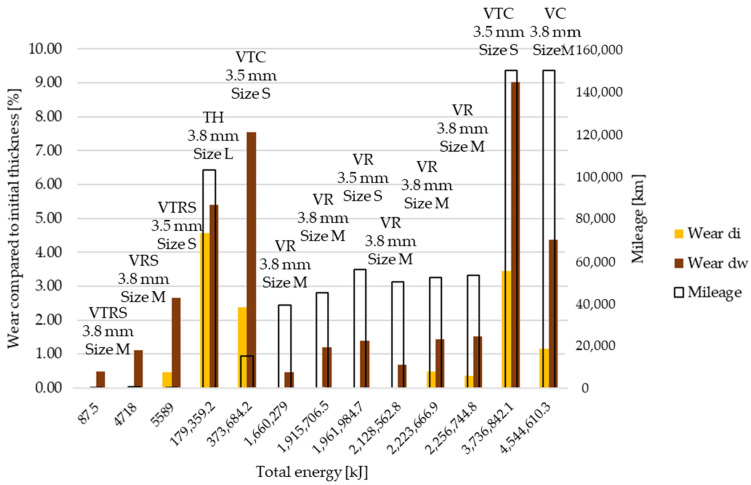
Relative wear values on inner (di) and working (dw) diameter with mileage along Joule scale.

Along the non-linear Joule scale two trends can be observed to be governing wear results after different test types, so measured data were separated accordingly: clutch killer and moderate usage. Figure 3a (clutch killer), Figure 3b (moderate) and Figure A1 in Appendix A illustrate these wear values along inner and working diameters respectively from Table 6 and Table A1. Dry clutch friction facing surfaces usually have a conicity due to the riveted joints between their cushioning spring segments and the side plates or hub flange of the disc. Therefore, during engagement, the facing cannot be considered flat; different wear values occur at different diameters and this continues during the lifetime of the friction surfaces. Moreover, the so-called tapering phenomenon influences contact parallelism as well: not only the facing, but also the flywheel and the pressure plate suffers remaining deformation, especially during rapid, repeated high energy loads. This can lead to changes in the relative conicity of mating surfaces, therefore closing the contact at inner diameters. Taking all that into consideration, wear–thickness loss–values were measured separately at two different diameters (so-called working, dw, and inner, di). These are the two diameters the pin-on-disc pin samples were to be cut from in our future study.

Comparing results, it can be concluded that the more total energy was applied to the system, the more wear was generated, with some exceptions, highlighting effects of other parameters from the system and conditions. In this paper all results are illustrated along activation energy values, though not in proportional scale.

Figure 3a shows the wear results of tests called ‘clutch killer’ load cases: when a trailer was attached to the vehicle (VTRS, VTC) or there were hill starts on purpose (VTRS, VRS), or a similar load case was simulated on a test bench (TH). Starting the vehicle upwards on a hill and/or with a trailer is considered to result in high energy loads due to longer slippage times, being the root cause of mentioned fast tapering that creates considerable wear at inner diameters. This can be seen when comparing Figure 3a inner diameter wear values with Figure 3b ‘di’ results called ‘moderate’ load cases that lacked the phenomenon.

The following conclusions can be drawn examining the data:

- Clutch killer tests created significantly higher wear values at inner diameters;
- TH wear result shows that mileage has a strong influence on wear besides applied energy, and a test condition such as the test bench (meaning more frequent actuation than in real life, or less time to cool down the system, using a closed test chamber with imitated heat flow and air suction, rapidly changing tapering) causes higher heat stress than in real life;
- On the other hand, TH test bench conditions produced less difference between inner and working diameter wear values;
- Analyzing wear values VTC showed wear conditions were severe even after lower mileage test runs and remained more intense even at high mileage runs: VTC tests run with trailer created engagements in non-optimal contact positions regarding the clutch friction surface, as dw wear was about three times higher than di wear regardless energy or mileage;
- The same phenomenon appeared in the VTRS test, causing a higher wear rate at lower mileage but with smaller diameter facings;
- Tests with actuation profiles happening among city traffic conditions (‘C’) meant an extra energy load on the system, similar to test bench conditions, as shorter cool down opportunities were present.

Examining Figure 3b, moderate test wear results highlight the effects of tests run on test tracks, where professional drove the vehicles (VR), and high mileage city tests, where the customer’s driving style determined the long-term wear values (VC), and the following can be seen:

- Moderate tests created significantly lower wear values at inner diameters;
- VR tests run in cars driven by professional drivers operating with presumably shorter shifting periods (slip) generated lower wear values;
- By more or less the same mileage smaller diameter, VR-tested facing (size S) showed higher wear values than greater diameter facings.
- VC results suggest that if the system was applied with loads from non-professional drivers, but for a long time (mileage), wear vales would be similarly moderate as those caused by professional drivers.

Overall, it can be stated, that:

- Wear values at di and dw diameter changed more or less according to the same trend, though working diameter material loss was higher considering relative values;
- Comparing inner and working radius values, the conical position of the facing at the beginning of the engagement was clear from lower wear values at the inner radius: di wear was always less than dw wear;
- Within the same test type category, the more energy applied, the higher the wear values;
- Though the more energy applied, the higher the wear losses, the exact amount was highly influenced by driving styles and the rapidity of energy load build-up at vehicle starts.

**Figure 4:**
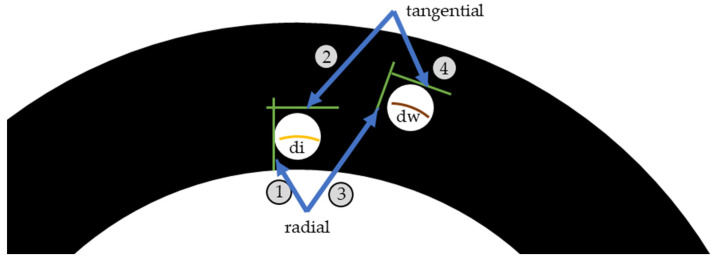
Definition of surface roughness measurement directions and diameters.

Examining surface roughness values of friction facings after the different tests and comparing the results with characteristics describing the surfaces of the unused friction material gives us another opportunity to judge the hypothetical test intensity scale. Therefore, measurements were carried out utilizing the MarSurf surface roughness measurement device equipped with a PHT 350 header. During these measurements measuring length was L_t_ = 4.8 mm and Ra, Rz and Rmax was determined. Figure 4 illustrates the diameters covered by the examination and the directions in which they were carried out.

Table A2, Table A3, Table A4 and Table A5 in Appendix B summarizes the results of surface roughness measurements. Values were averaged from measured data. Deviation is the calculated sigma (σ) based on 8 repeated measurements. Reference value is from a facing after production steps were finished.

Table A2 contains values measured at the inner diameter in radial direction (arrow Nr 1 highlights it in Figure 4), whereas Table A3 represents results at the same diameter but in tangential direction, as arrow Nr 2 illustrates in Figure 4. Working diameter values divided on the same principle are contained in Table A4 and Table A5 in radial and tangential directions, respectively, as Figure 4 arrows Nr 3 and 4 highlight. Table A6 in Appendix B contains relative surface roughness values compared to the reference results.

**Figure 5:**
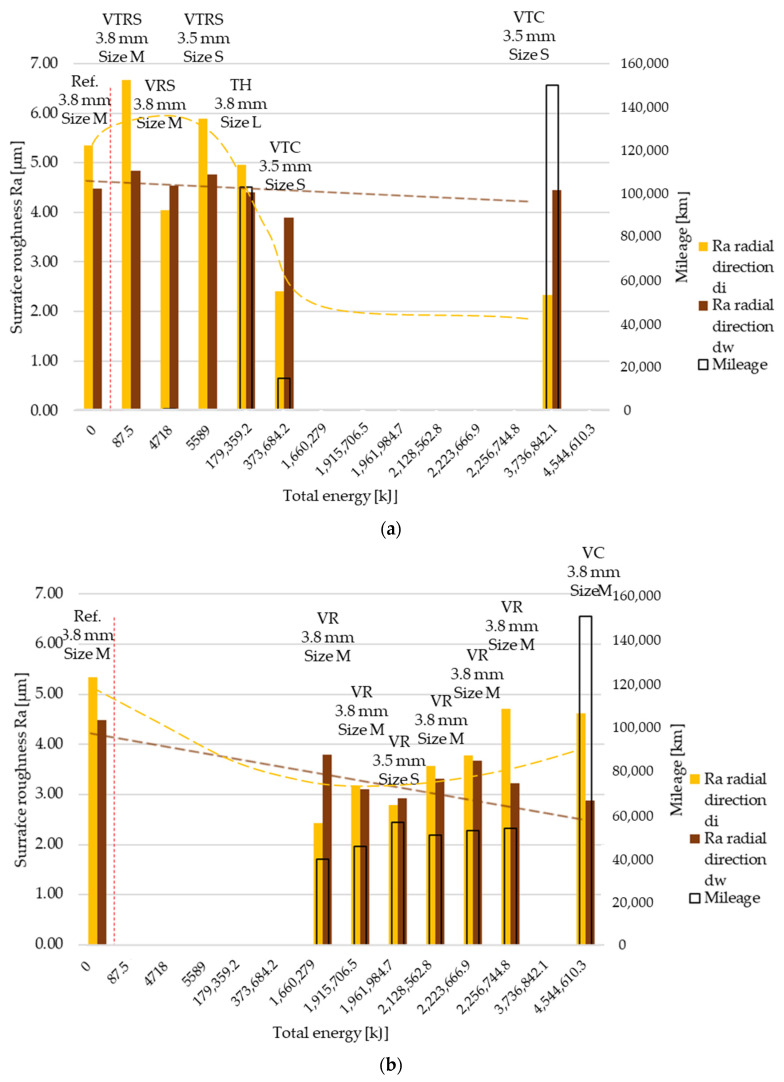
(**a**) Ra surface roughness values, radial direction–trend 1: clutch killer tests. (**b**) Ra surface roughness values, radial direction–trend 2: moderate tests.

Ra surface roughness values at inner (di) and outer (dw) diameter in radial direction are illustrated in Figure 5a,b, continuing the separation of trends introduced by wear results. Reference values are highlighted on the left side of the red dotted line. Trend 1 results suggest that tests where sudden, high-energy impulses rather than continuous energy distribution describe the load case result in less smooth surfaces. Trend 1 results above reference values suggest that surfaces were torn up each time engagement happened, leading to rougher surfaces. Note that radial direction measurements cross the surface along different diameters. Along this direction, load characteristic can change significantly.

**Figure 6:**
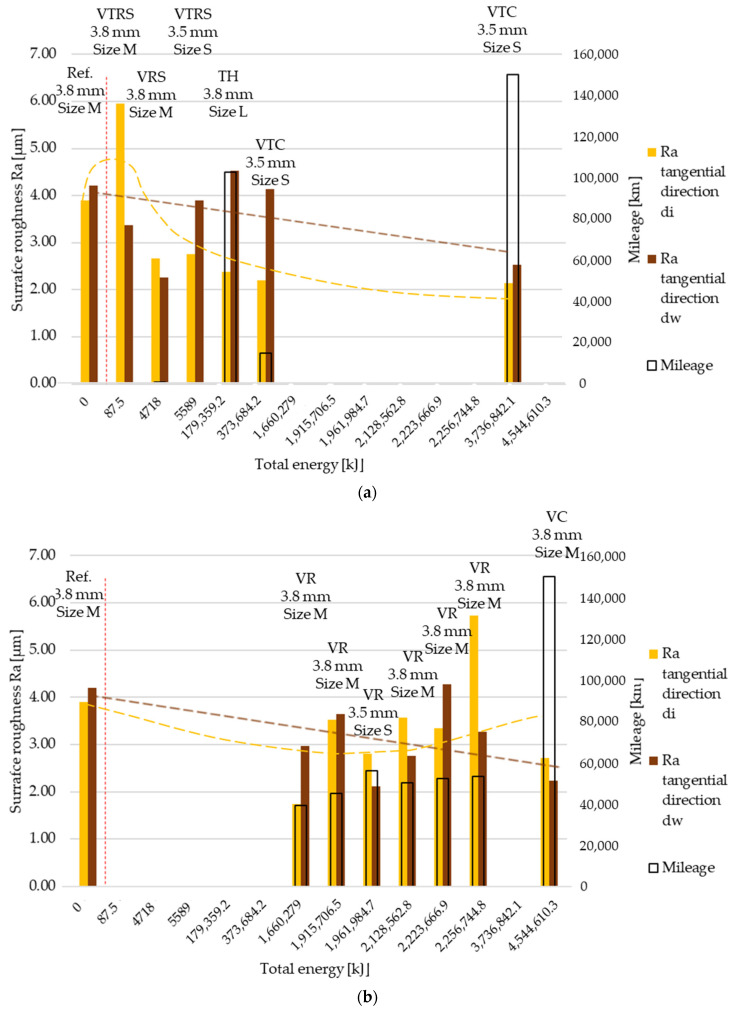
(**a**) Ra surface roughness values, tangential direction–trend 1: clutch killer tests. (**b**) Ra surface roughness values, tangential direction–trend 2: moderate tests.

Tangential direction Ra surface roughness distribution is illustrated in Figure 6a,b with the trend separation kept. Comparing ‘a’ and ‘b’ figures, the difference between the trends was not as significant as that in radial direction results.

**Figure 7:**
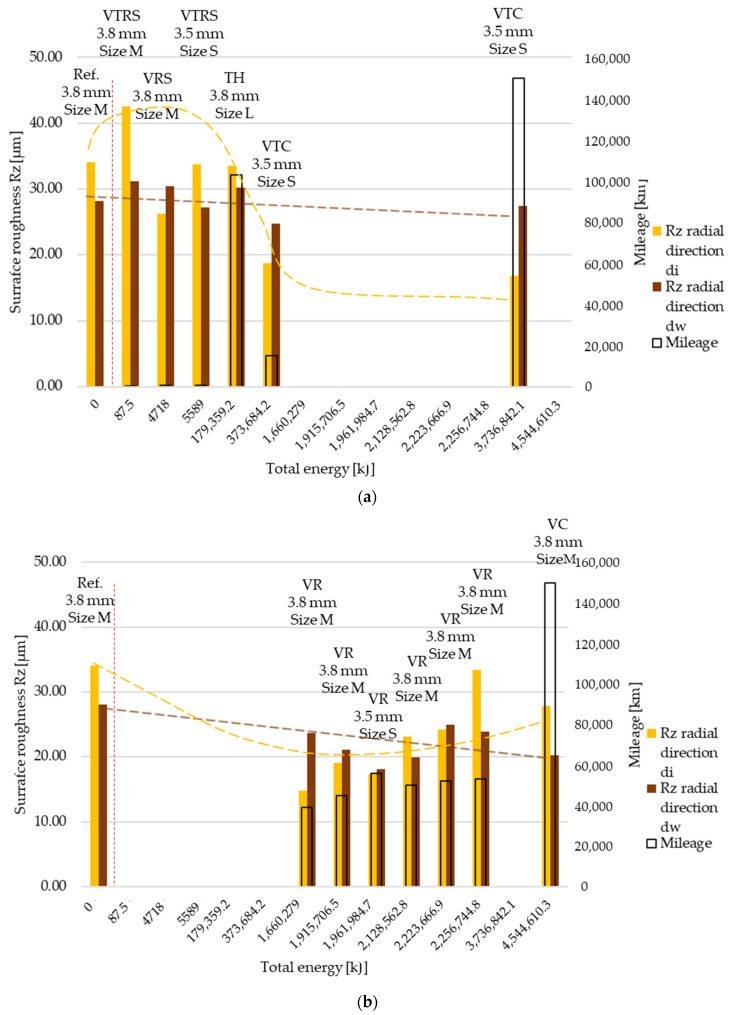
(**a**) Rz surface roughness values, radial direction–trend 1: clutch killer tests. (**b**) Rz surface roughness values, radial direction—trend 2: moderate tests.

**Figure 8:**
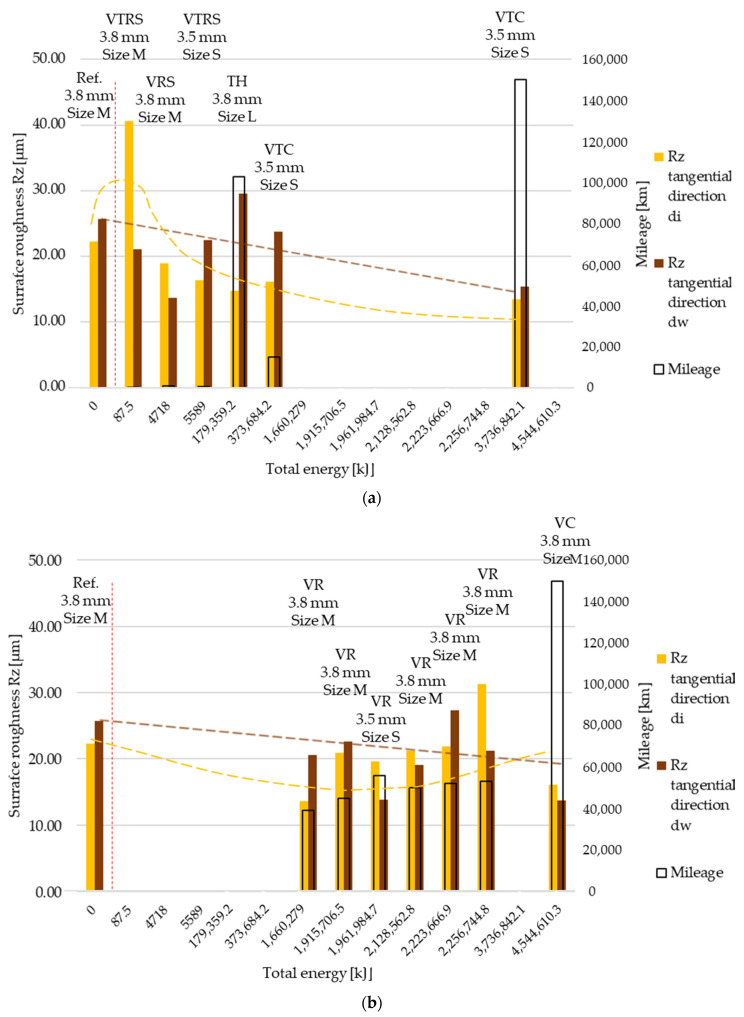
(**a**) Rz surface roughness values, tangential direction–trend 1: clutch killer tests. (**b**) Rz surface roughness values, tangential direction–trend 2: moderate tests.

Measured then separated Rz surface roughness values are illustrated in Figure 7 and Figure 8, in radial and tangential directions, respectively. Trends and relative values did not differ much from Ra measurement results.

**Figure 9:**
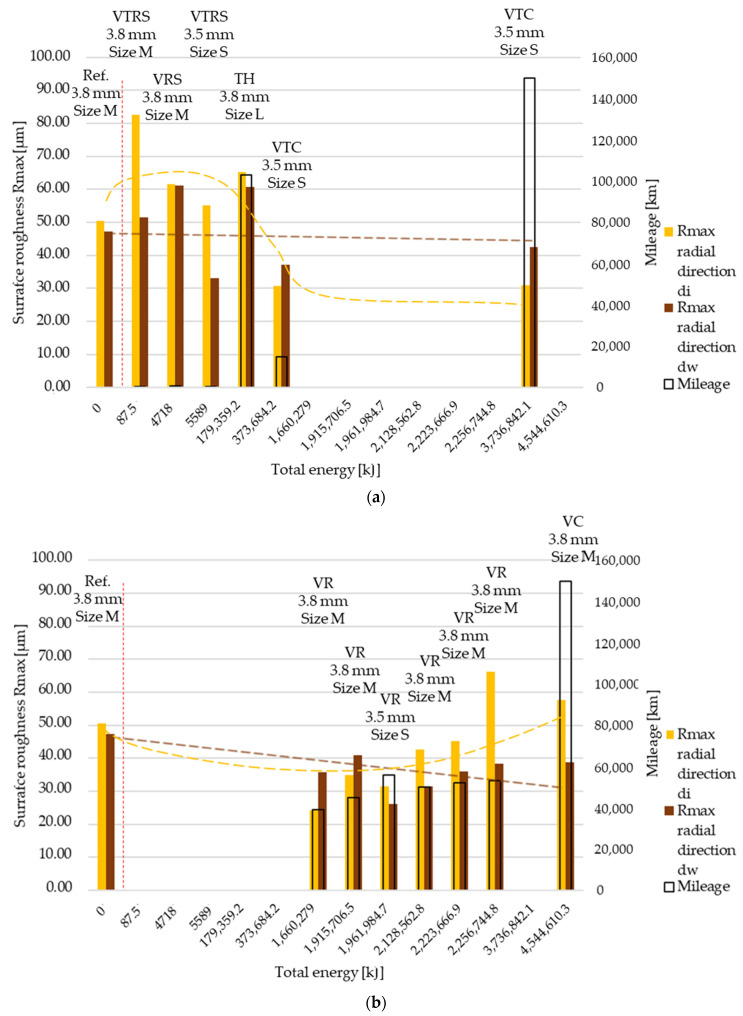
(**a**) Rmax surface roughness values, radial direction–trend 1: clutch killer tests. (**b**) Rmax surface roughness values, radial direction–trend 2: moderate tests.

**Figure 10:**
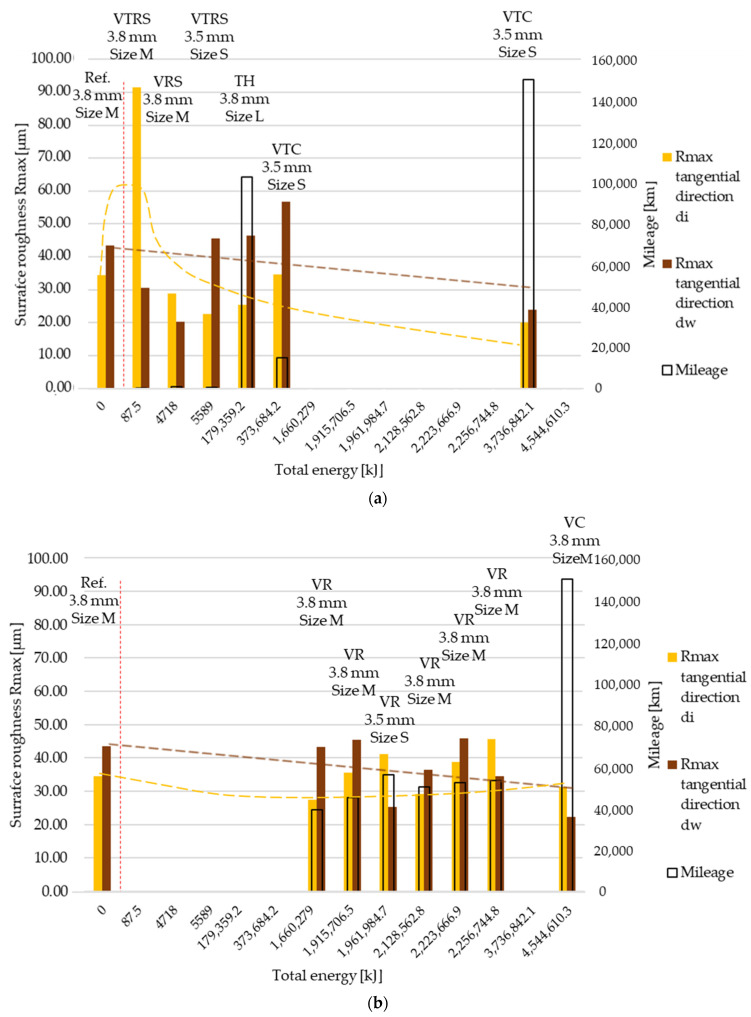
(**a**) Rmax surface roughness values, tangential direction–trend 1: clutch killer tests. (**b**) Rmax surface roughness values, tangential direction–trend 2: moderate tests.

Rmax results are illustrated in Figure 9 and Figure 10. As before, ‘a’ corresponds to trend 1 originated from wear values, ‘b’ to trend 2. Results represent measurement data in radial and tangential directions. Trend 1 results compared to reference value clearly highlight the high energy, long slippage-induced surface tear-up phenomena suspected from the Ra and Rz trend 1 roughness measurements.

**Figure 11:**
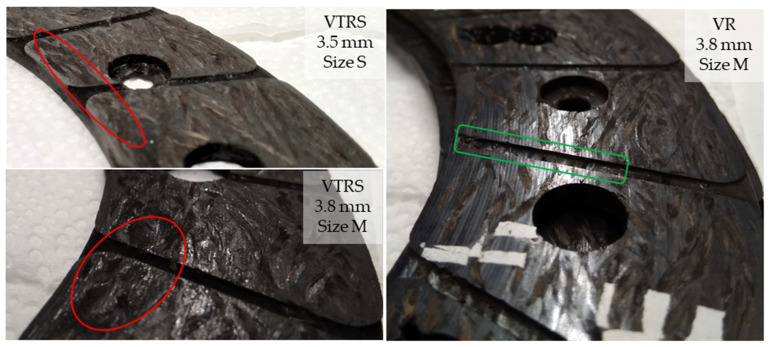
Visual inspection of surfaces after trend 1 (VTRS) and trend 2 (VR) tests.

Examining the surfaces by visual inspection, the differences at inner diameter were clear. Figure 11 compares two facing surfaces after a VTRS test with the consequences of a VR test. As illustrated, VTRS surfaces showed deeper grooves with darker color, indicating thermal damage of fibers on the surface. This could be a result of the rapidly applied energy loads in a heavy vehicle and/or a long slippage time, whereas a VR test resulted in a more evenly worn surface with regular grooves.

Radial and tangential direction surface roughness values decreased on the working diameter along the increasing applied energy scale, with some exceptions due to different parameters governing their application. Trends observed from wear results were present and Rmax values highlight them more significantly than Ra or Rz results: clutch killer low energy and mileage test effects were even more significant with increased Rmax values on both inner and working diameters in radial and axial direction, proving the theory of torn-up surfaces.

There was a difference between radial and tangential Ra results regarding the amount of surface energy value from which roughness started to decrease along the Joule scale. This could be the result of the fact that radial measurement crosses through diameters with slightly different load and wear conditions.

The following can also be seen from the measurements:

- Inner diameter results highlight the presumably present non-parallel position of mating surfaces due to tapering: test results with trailer produced higher roughness values on inner diameter than on outer diameter in low energy and mileage tests (VTRS), while the opposite was true after the initial running-in phase (see VTC results after 10,000 km and 150,000 km);
- The inner diameter trend showed more deviations that could be caused by the tapering occurring during multiple repeated shifts during which deformation influenced contact areas, so that it resulted in drastically different wear at different diameters;
- VR test results produced the lowest roughness values;
- Within VR results, small size facing roughness values were the smoothest;
- After VR tests, where professional drivers were operating the clutch, di and dw results were not that different, highlighting the possibility of less slip and deformation (remaining conicity) caused by heat stress;
- However, working diameter Ra roughness values did not decrease linearly along the Joule scale: in low mileage and energy tests the trend was decreasing, so similar mileage tests resulted in similar Ra values, and smaller size facings were characterized by lower roughness values after energy and mileage tests similar to those on larger size facings;
- Radial direction Rz surface roughness values slightly decreased on the working diameter along the increasing applied energy scale;
- Inner diameter Rz values followed the same trend that describes inner diameter Ra characteristics;
- Tangential direction Rz surface roughness values slightly decreased as well on the working diameter along the increasing applied energy scale;
- Working diameter radial direction Rmax values more or less followed the other dw surface roughness characteristics, while inner diameter values did not show as much deviance from dw values as other features;
- The same conclusions can be drawn from tangential direction Rmax values.

**Figure 12:**
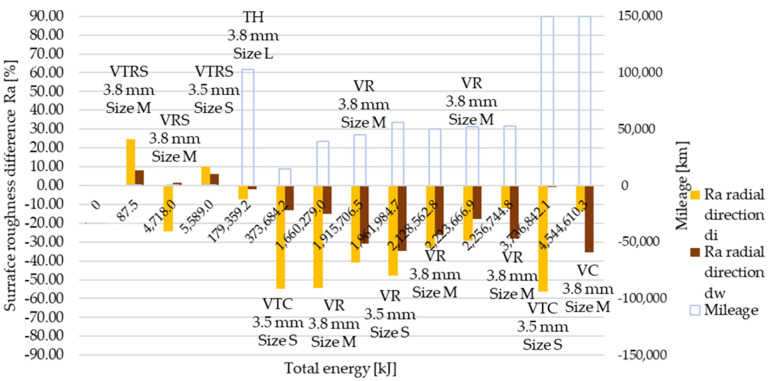
Deviation of Ra surface roughness values from initial conditions, radial direction.

**Figure 13:**
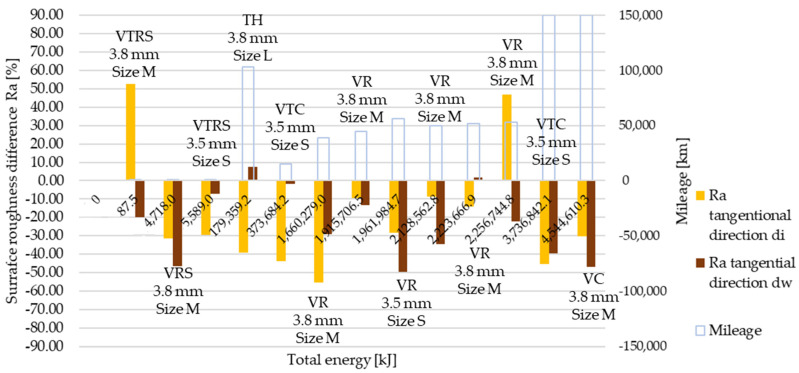
Deviation of Ra surface roughness values from initial conditions, tangential direction.

**Figure 14:**
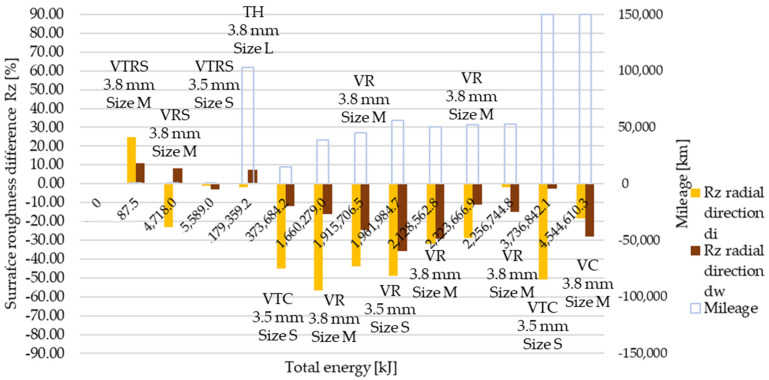
Deviation of Rz surface roughness values from initial conditions, radial direction.

**Figure 15:**
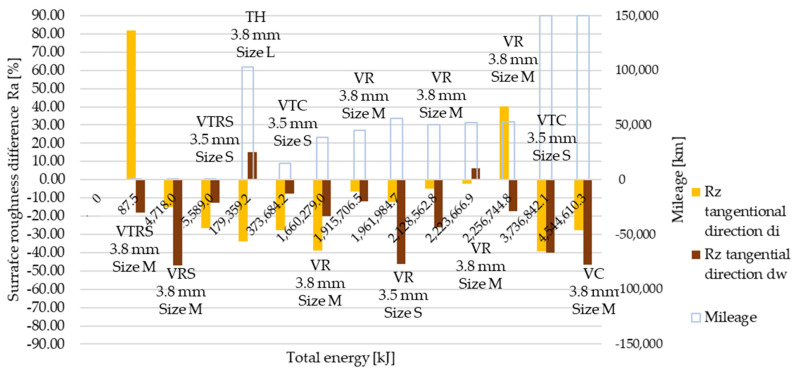
Deviation of Rz surface roughness values from initial conditions, tangential direction.

**Figure 16:**
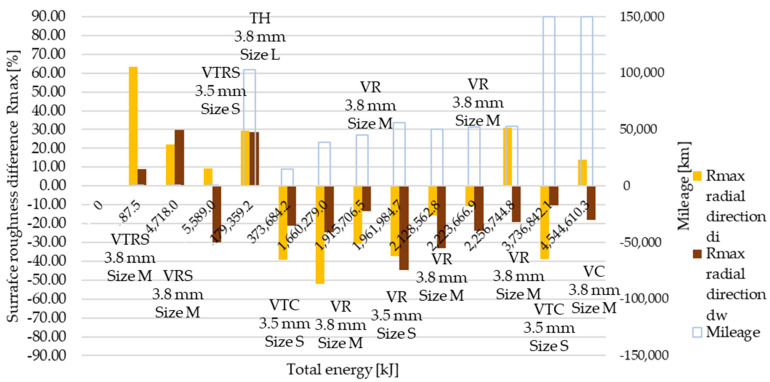
Deviation of Rmax surface roughness values from initial conditions, radial direction.

**Figure 17:**
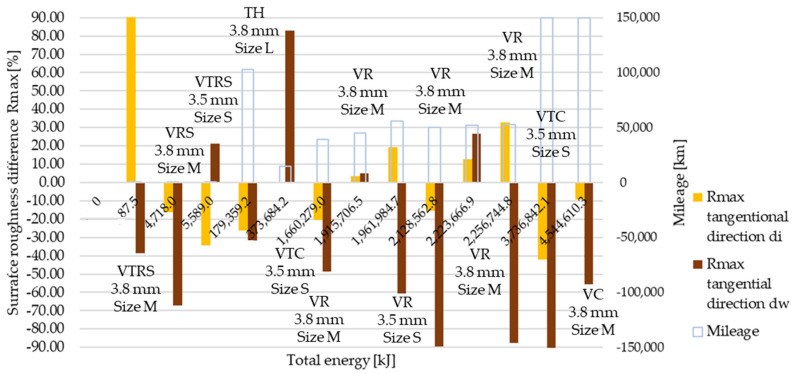
Deviation of Rmax surface roughness values from initial conditions, tangential direction.

Table A6 details and Figure 12, Figure 13, Figure 14, Figure 15, Figure 16 and Figure 17 illustrate how the tests transformed the surface characteristics of the friction facings after the different tests on different diameters and in different directions. Since the base of the comparison was always the unused facing surface roughness, the trend of results resembles the trend seen by absolute values.

Detailing surface roughness deviation as a percentage, the following can be seen from the Ra, Rz and Rmax results:

- The fact that tapering and deformation induced higher deviation at inner diameter results in drastically different wear at different diameters is clearly highlighted;
- Comparing Ra difference values in radial direction highlights more intensive surface roughness value changes by inner diameter than working diameter;
- In the tangential direction, low energy or mileage can cause deviations from the trend along the Joule-scale;
- Rz deviation values produced similar results; both clutch killer and moderately operated test results had a good correlation with Ra trends at inner and outer diameter, respectively, considering both radial and tangential directions, even with percentage values taken into account, though tangential direction deviations on the inner diameter were relatively smaller.
- Low energy and mileage results can lead to increased Rmax values on the inner diameter both in radial and tangential direction, as VTRS results suggest;
- Only test bench TH investigation increased the working diameter tangential Rmax value significantly, highlighting the fact that test rig results do not necessarily recreate real conditions-induced results regarding all aspects.

As clutch killer tests increased wear along the Joule scale on the working diameter, so the surfaces became smoother in radial and tangential direction as well regarding Ra. However, as TH results highlight, high mileage or lack of proper cooling (test bench) can lead to increasing tangential Ra surface roughness at the working diameter, probably due to rapid thermal overloads at certain spots tearing up the surface and causing remaining macroscopic deformations. Additionally, a steeper wear trend is paired with less decreasing Ra values in the radial direction.

Moderate tests led to less wear on the working diameter that was paired with decreasing Ra surface roughness in both directions along the activation energy scale, though deviations could be observed when there was significant inner diameter wear that also led to increased inner diameter Ra surface roughness both in radial and tangential directions. Among the test condition results, smaller size facings suffered higher wear that was paired with lower tangential and radial Ra surface roughness.

Inner diameter results after clutch killer tests highlight the presumably present non-parallel position of mating surfaces due to tapering: test results with a trailer produced higher radial Ra roughness values on the inner diameter than on the outer diameter in low energy and mileage tests (VTRS), while the opposite can be stated after the initial running-in phase (VTC after 10,000 km versus 150,000 km).

Moderate tests caused less wear at the inner diameter, hence the hardly changing Ra values in both directions. However, when there was wear, Ra could increase above the reference level, as mentioned.

## 4. Conclusions

Tribological aspects of fiber-reinforced hybrid composite dry friction clutch facings were examined in a novel way: after different, mainly non-laboratory automotive tests, that were characterized by different surface activation energy values. Tests were carried out according to automotive industry best practice, then transmission systems were disassembled so friction facings were able to be examined regarding their thickness loss and surface roughness to characterize tribologically induced changes of the friction surface. Thickness loss–wear and changes of microgeometry characteristics–and surface roughness values Ra, Rz and Rmax from two different diameters (inner and working) were compared with the aim of highlighting the main governing parameters influencing these values during clutch facing lifetime.

Wear values showed double increasing trends based on test types: two groups in the analyzed tests could be sorted into: ‘clutch killer’ and ‘moderate’ applications. The former consisted of tests during which a trailer was attached to the vehicle (VTRS, VTC) or there were hill starts on purpose (VTRS, VRS), or a similar load case was simulated on a test bench (TH). Starting the vehicle upwards on a hill and/or with a trailer led to high energy loads due to longer slippage times, being the root cause of so-called tapering (strain-induced misalignment of mating friction surfaces) that creates considerable wear–above 0.1 mm–also at inner diameters. The other group was formed by tests in which professional drivers were operating the clutch, mainly on test tracks, or a customer drove the car for more than a year with high mileage among mainly constant conditions.

‘Clutch killer’ inner diameter wear results followed an increasing trend along the Joule scale. The relative material loss was influenced by the mileage and test conditions: 1% higher wear was generated by 90% higher mileage; another 1% increment could be caused by insufficient cooling time or test bench-specific conditions.

Increased inner diameter Ra results paired with low or zero thickness loss suggested torn-up surfaces due to rapid, low mileage applications of the clutch. The highest relative roughness decrease was caused by the tests resulting in one of the highest relative thickness losses. The same trend describes ‘clutch killer’ inner diameter Rz values in the radial direction. Rmax values in the radial direction at the inner diameter differed from only those of lower energy or lowest mileage tests: roughness increased by 20–60%, highlighting surface tear. Overall, tests run with a trailer and in city conditions resulted in more than 2% thickness loss and a 40–50% roughness decrease.

Tangential direction Ra values at inner diameter followed more closely the trends seen from wear results with more intensive roughness decrease paired with higher thickness loss, with only the lowest energy and mileage tests resulting in increasing roughness. Changes in Rz values followed the same trend. Rmax results highlight the low energy roughness increase as well, but differed from other values of the 15,000 km VTC results.

‘Moderate’ inner diameter wear results followed a less steep increasing trend compared to ‘clutch killer’ values along the Joule scale. The highest energy and mileage application led to the highest thickness loss: more than 0.3 mm after 140,000 km and more than 3.7 GJ.

Inner diameter radial direction Ra and Rz values decreased less and less along the non-proportional Joule scale with Rmax values resulting in a roughness increase in the highest energy test in this group.

In the tangential direction, changes of the three roughness values followed the same trend, but lacking wear, no correlation can be drawn. The Ra trend showed less moderate changes.

Comparing ‘clutch killer’ and ‘moderate’ roughness result trends at the inner diameter, by the former, tangential results followed more closely wear result trends, while for the latter, radial value trends behaved following an inverse trend regarding percentage change.

‘Clutch killer’ working diameter wear results followed an increasing trend along the Joule scale. Mileage or test-specific conditions did not have such influence that it was present among inner diameter results.

Working diameter ‘clutch killer’ Ra results in the radial direction showed an increase in lower energy tests. Rz and Rmax results showed sensitivity to facing size: small (S) facings produced significantly decreased roughness, while large (L) and medium (M) size facings had increased roughness values. Rmax results showed the highest deviations among roughness values in the radial direction.

In the tangential direction, none of the roughness value change trends followed wear behavior, but Ra and Rz showed sensitivity to test conditions, resulting in roughness increase after the TH test.

‘Moderate’ working diameter wear results followed a less steep increasing trend compared to ‘clutch killer’ values along the Joule scale, just like those of the inner diameter.

Radial direction working diameter roughness value trends were very similar to inner diameter trends with Rmax correlating better with the other two roughness results along the energy scale.

The three tangential direction working diameter roughness trends were the same, with the small size facings producing the highest decrease in Ra and Rz roughness values as they had higher thickness loss compared to medium size facings with similar surface activation energy.

Comparing the two different trends from the roughness point of view, working diameter value trends did not show the difference between tangential and radial behavior that was present among inner diameter results.

Different activation energy-induced changes in surface microgeometry of automotive dry clutch facings are heavily influenced by operating parameters such as mileage and driving style and also affected by facing size. The hypothesis that more activation energy results in an increased amount of wear and smoother surfaces is only right if those conditions and deviation in behavior between different diameters is considered.

Future investigations will further examine tribological behavior and its effects during the component’s lifetime via pin-on-disc tests. The final aim is to extend the prediction capability of currently utilized contact models coupled with the thermomechanical and tribomechanical analyses considered.

## Figures and Tables

**Table 1:** Material properties of the hybrid composite friction facing.

Property	Matrix	Fiber Reinforcement	Whole Material
Young modulus [MPa]	4290	27,300	direction dependent
Poisson’s ratio [-]	0.38	0.2	direction dependent
Shear modulus [MPa]	1290	11,380	direction dependent
Thermal conductivity coefficient [W/(m∙K)]	-	-	0.398

**Table 2:** Automotive industry tests ensuring dry clutch facing quality during its lifetime.

Test	Application	Track	Acceleration	Length	Shifts	Conditions	Driver
Test bench-Highway [TH]	Test bench	simulating highway in laboratory	60–70% max. acceleration, 30–40% country side, 1–2% idle				
Vehicle City [VC]	Vehicle	street	average	1 year	frequent shifting	high mileage	average customer
Vehicle + Trailer–City [VTC]	Vehicle + trailer	street	average	1 year	frequent shifting	high mileage	average customer
Vehicle-Ring (test track) [VR]	Vehicle	test track	acceleration under full load		frequent shifting (2nd–6th gear)	max. rotational velocity, thermal peaks	professional drivers
Vehicle + Trailer-Ring with Start on Hill [VTRS]	Vehicle + trailer	test track + hill start	hill start with warm up and cool down, trailer, 1500–2800 rpm			up to 370 °C peaks	
Vehicle-Ring with Start on Hill [VRS]	Vehicle	test track + hill start	hill start before horizontal			300–400 °C	

**Table 3:** Energy during different actuation profiles: calculated average applied energy per shift.

App.	Actuation Profile			City	City + Hill	Country	High-Way	Traffic Jam	Launch 12% Steep	Launch 16% Steep	Launch 20% Steep
Vehicle	Launch	Engine speed	[RPM]	1400	1400	1400	1400	2000	1500	1600	1900
		Energy	[kJ]	4.9	4.9	4.9	4.9	10.6	11.3	16.8	23.8
	Shift +	Energy	[kJ]	0.2	0.2	0.2	0.2	−	−	−	−
	Shift −	Energy	[kJ]	0.8	0.8	0.8	0.8	−	−	−	−
Vehicle + trailer	Launch	Engine speed	[RPM]	1400	1400	1400	1400	2000	2000	−	−
		Energy	[kJ]	4.9	4.9	4.9	4.9	10.6	23.6	−	−
	Shift +	Energy	[kJ]	0.2	0.2	0.2	0.2	−	−	−	−
	Shift −	Energy	[kJ]	0.8	0.8	0.8	0.8	−	−	−	−

**Table 4:** Intensity scale of preliminary tests based on average surface activation energy per kilometer per area unit.

Test Code		TH	VRS	VRTS	VC	VTC	VR
Test intensity	[J/km/cm^2^]	6.6	25	55	120	152	170
		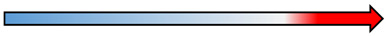					

**Table 5:** Matrix of automotive tests: test samples along mileage and test intensity axes.

Samples from Automotive Tests				Mileage [1000 km]											
Test		Facing Size		0.006	0.5	0.8	15	39	45	50	52	53	56	102.9	150
Name	Test Intensity [J/km/cm^2^]	Diameters Outer/Inner [mm]	Thickness [mm]												
VRS	6.6	228/160	3.5												
		240/160	3.8			VRS-0008M									
		240/155													
VTRS	25	228/160	3.5		VTRS-0005S										
		240/160	3.8	VTRS-000M											
		240/155													
TH	55	228/160	3.5												
		240/160	3.8												
		240/155												TH-103L	
VC	120	228/160	3.5												
		240/160	3.8												VC-150M
		240/155													
VTC	128	228/160	3.5				VTC-015S								VTC-150S
		240/160	3.8												
		240/155													
VR	170	228/160	3.5										VR-056S		
		240/160	3.8					VR-039M	VR-045M	VR-050M	VR-052M	VR-053M			
		240/155													

**Table 6:** Wear values of facings after different automotive tests.

Test	Specimen	Mileage	Total Energy	Wear, di	Deviation, σ	Wear, dw	Deviation, σ
		[km]	[kJ]	[mm]	[mm]	[mm]	[mm]
VTRS	VTRS-000M	6	87.5	0	0	0.02	0.018
VRS	VRS-0008M	793	4718	0	0	0.04	0.029
VTRS	VTRS-0005S	496	5589	0.02	0.017	0.10	0.060
TH	TH-103L	102,900	179,359.2	0.16	0.12	0.19	0.087
VTC	VTC-015S	15,000	373,684.2	0.09	0.048	0.29	0.057
VR	VR-039M	39,000	1,660,279.0	0	0	0.02	0.019
VR	VR-045M	45,000	1,915,706.5	0	0	0.05	0.042
VR	VR-056S	56,000	1,961,984.7	0	0	0.05	0.044
VR	VR-050M	50,000	2,128,562.8	0	0	0.03	0.020
VR	VR-052M	52,000	2,223,666.9	0.02	0.01	0.05	0.018
VR	VR-053M	53,000	2,256,744.8	0.01	0.0053	0.06	0.018
VTC	VTC-150S	150,000	3,736,842.1	0.12	0.045	0.32	0.097
VC	VC-150M	150,000	4,544,610.3	0.04	0.015	0.17	0.071

## Appendix A

**Table A1:** Relative wear values of facings from the different automotive tests.

Test	Specimen	Mileage	Total Energy	Wear di	Wear dw
		[km]	[kJ]	[%]	[%]
VTRS	VTRS-000M	6	87.5	0.00	0.50
VRS	VRS-0008M	793	4718	0.00	1.11
VTRS	VTRS-0005S	496	5589	0.46	2.66
TH	TH-103L	102,900	179,359.2	4.57	5.40
VTC	VTC-015S	15,000	373,684.2	2.37	7.55
VR	VR-039M	39,000	1,660,279.0	0.00	0.47
VR	VR-045M	45,000	1,915,706.5	0.00	1.21
VR	VR-056S	56,000	1,961,984.7	0.00	1.39
VR	VR-050M	50,000	2,128,562.8	0.00	0.68
VR	VR-052M	52,000	2,223,666.9	0.49	1.43
VR	VR-053M	53,000	2,256,744.8	0.37	1.53
VTC	VTC-150S	150,000	3,736,842.1	3.46	9.03
VC	VC-150M	150,000	4,544,610.3	1.16	4.36

## Appendix B

**Table A2:** Summary of the surface roughness values before (reference) and after different tests: inner diameter, radial direction.

Test	Specimen	Mileage	Spot	Inner Diameter, di					
			Total Energy	Ra Rad	Deviation, σ	Rz Rad	Deviation, σ	R Max Rad	Deviation, σ
		[km]	[kJ]	[µm]	[µm]	[µm]	[µm]	[µm]	[µm]
Ref.	X-000L	0	0	5.35	0.51	34.06	4.45	50.49	10.17
VTRS	VTRS-000M	6	87.5	6.67	0.28	42.56	2.69	82.49	3.11
VRS	VRS-0008M	793	4718	4.04	0.58	26.23	4	61.61	25.06
VTRS	VTRS-0005S	496	5589	5.89	0.61	33.74	2.49	55.23	6.1
TH	TH-103L	102,900	179,359.2	4.96	0.03	33.50	4.45	65.25	32.2
VTC	VTC-015S	15,000	373,684.2	2.41	0.19	18.75	4	30.61	9.99
VR	VR-039M	39,000	1,660,279.0	2.43	0.33	14.83	3.1	24.24	5.91
VR	VR-045M	45,000	1,915,706.5	3.17	0.83	19.11	7.03	34.95	10.4
VR	VR-056S	56,000	1,961,984.7	2.80	0.56	17.41	2.23	31.56	3.72
VR	VR-050M	50,000	2,128,562.8	3.57	0.68	23.12	3.51	42.60	8.31
VR	VR-052M	52,000	2,223,666.9	3.79	0.41	24.25	5.87	45.12	19.89
VR	VR-053M	53,000	2,256,744.8	4.72	0.48	33.44	6.26	66.20	21.51
VTC	VTC-150S	150,000	3,736,842.1	2.34	0.2	16.80	0.65	30.85	0.02
VC	VC-150M	150,000	4,544,610.3	4.63	1.33	27.82	9.27	57.58	29.56

**Table A3:** Summary of the surface roughness values before (reference) and after different tests: inner diameter, tangential direction.

Test	Specimen	Mileage	Spot	Inner Diameter, di					
			Total Energy	Ra Tng	Deviation, σ	RZ TNG	Deviation, σ	R Max Tng	Deviation, σ
		[km]	[kJ]	[µm]	[µm]	[µm]	[µm]	[µm]	[µm]
Ref.	X-000L	0	0	3.90	1.44	22.29	7.46	34.46	15.85
VTRS	VTRS-000M	6	87.5	5.96	0	40.58	0	91.55	0
VRS	VRS-0008M	793	4718	2.67	0.63	18.99	4.57	28.88	3.9
VTRS	VTRS-0005S	496	5589	2.76	0.25	16.35	1.55	22.60	4.39
TH	TH-103L	102,900	179,359.2	2.37	0.55	14.71	1.98	25.38	9.69
VTC	VTC-015S	15,000	373,684.2	2.20	1.12	16.13	7.36	34.59	15.13
VR	VR-039M	39,000	1,660,279.0	1.75	1.06	13.59	6.36	27.35	15.87
VR	VR-045M	45,000	1,915,706.5	3.53	0.14	20.85	0.89	35.68	9.21
VR	VR-056S	56,000	1,961,984.7	2.80	0.78	19.56	8.51	41.15	18.97
VR	VR-050M	50,000	2,128,562.8	3.57	1	21.19	5.39	29.12	7.85
VR	VR-052M	52,000	2,223,666.9	3.35	0.41	21.82	2.45	38.90	10.96
VR	VR-053M	53,000	2,256,744.8	5.73	1.46	31.27	11.45	45.70	5.8
VTC	VTC-150S	150,000	3,736,842.1	2.14	0.75	13.50	1.04	20.00	0.56
VC	VC-150M	150,000	4,544,610.3	2.71	1.62	16.09	10.04	31.20	21.38

**Table A4:** Summary of the surface roughness values before (reference) and after different tests: outer diameter, radial direction.

Test	Specimen	Mileage	Spot	Working Diameter, dw					
			Total Energy	Ra Rad	Deviation, σ	Rz rad	Deviation, σ	R Max Rad	Deviation, σ
		[km]	[kJ]	[µm]	[µm]	[µm]	[µm]	[µm]	[µm]
Ref.	X-000L	0	0	4.48	1.42	28.13	9.34	47.26	20.42
VTRS	VTRS-000M	6	87.5	4.84	1.39	31.20	11.04	51.51	11.75
VRS	VRS-0008M	793	4718	4.55	0.20	30.40	3.17	61.26	4.01
VTRS	VTRS-0005S	496	5589	4.76	0.49	27.26	5.44	33.17	5.18
TH	TH-103L	102,900	179,359.2	4.40	1.03	30.21	8.41	60.72	15.01
VTC	VTC-015S	15,000	373,684.2	3.90	0.02	24.75	0.62	37.20	1.19
VR	VR-039M	39,000	1,660,279.0	3.80	0.08	23.64	0.29	35.69	0.96
VR	VR-045M	45,000	1,915,706.5	3.10	0.58	21.15	3.45	40.85	0.48
VR	VR-056S	56,000	1,961,984.7	2.93	0.68	18.10	4.12	26.14	4.13
VR	VR-050M	50,000	2,128,562.8	3.31	0.50	19.93	2.67	31.57	6.20
VR	VR-052M	52,000	2,223,666.9	3.68	0.20	24.96	3.13	36.07	5.41
VR	VR-053M	53,000	2,256,744.8	3.22	0.00	23.93	0.00	38.25	0.00
VTC	VTC-150S	150,000	3,736,842.1	4.45	1.18	27.37	7.00	42.45	18.57
VC	VC-150M	150,000	4,544,610.3	2.88	0.69	20.26	5.16	38.71	18.38

**Table A5:** Summary of the surface roughness values before (reference) and after different tests: outer diameter, tangential direction.

Test	Specimen	Mileage	Spot	Working Diameter, dw					
			Total Energy	Ra Tng	Deviation, σ	Rz Tng	Deviation, σ	R Max Tng	Deviation, σ
		[km]	[kJ]	[µm]	[µm]	[µm]	[µm]	[µm]	[µm]
Ref.	X-000L	0	0	4.21	1.27	25.70	7.15	43.55	17.41
VTRS	VTRS-000M	6	87.5	3.37	1.58	21.09	8.15	30.67	10.72
VRS	VRS-0008M	793	4718	2.25	0.21	13.67	2.53	20.21	5.72
VTRS	VTRS-0005S	496	5589	3.90	1.2	22.48	3.6	45.58	21.14
TH	TH-103L	102,900	179,359.2	4.53	0.85	29.58	10.5	46.53	11.91
VTC	VTC-015S	15,000	373,684.2	4.13	0.98	23.79	1.79	56.84	31.88
VR	VR-039M	39,000	1,660,279.0	2.97	0.17	20.54	3.6	43.27	8.92
VR	VR-045M	45,000	1,915,706.5	3.64	1.81	22.64	5.15	45.41	18.26
VR	VR-056S	56000	1,961,984.7	2.12	0.74	13.81	0.93	25.20	6.83
VR	VR-050M	50,000	2,128,562.8	2.76	0.45	19.03	4.6	36.36	1.82
VR	VR-052M	52,000	2,223,666.9	4.28	0.45	27.28	5.11	45.85	22.06
VR	VR-053M	53,000	2,256,744.8	3.27	1.3	21.24	3.79	34.51	2.18
VTC	VTC-150S	150,000	3,736,842.1	2.54	0.33	15.42	3.25	23.99	0.72
VC	VC-150M	150,000	4,544,610.3	2.23	1.05	13.73	6.18	22.28	7.72

**Table A6:** Deviation of tested surface roughness values of facings from the initial surface roughness characteristics.

Test	Specimen	Mileage	Spot	Inner Diameter						Working Diameter					
			Total Energy	dRa Rad	Drz Rad	Dr Max Rad	dRa Tng	dRz Tng	dR Max Tng	dRa rad	dRz Rad	dR Max Rad	dRa Tng	dRz Tng	dR Max Tng
		[km]	[kJ]	%	%	%	%	%	%	%	%	%	%	µm	µm
Reference	X-000M	0	0	0	0	0	0	0	0	0	0	0	0	0	0
VTRS	VTRS-000M	6	87.5	24.74	24.95	63.4	52.59	82.05	165.66	8.08	10.93	9	−19.80	−17.93	−38.45
VRS	VRS-0008M	793	4718	−24.37	−23.00	22.02	−31.62	−14.78	−16.19	1.43	8.08	29.62	−46.41	−46.83	−67.13
VTRS	VTRS-0005S	496	5589	10.16	−0.92	9.39	−29.35	−26.66	−34.43	6.16	−3.08	−29.82	−7.23	−12.53	21.40
TH	TH-103L	102,900	179,359.2	−7.19	−1.65	29.24	−39.18	−34.01	−26.34	−1.85	7.43	28.48	7.58	15.09	−31.60
VTC	VTC-015S	15,000	373,684.2	−54.99	−44.94	−39.37	−43.67	−27.63	0.37	−13.03	−12.00	−21.29	−1.79	−7.45	83.07
VR	VR-039M	39,000	1,660,279.0	−54.58	−56.46	−51.99	−55.23	−39.03	−20.65	−15.20	−15.93	−24.47	−29.31	−20.10	−48.76
VR	VR-045M	45,000	1,915,706.5	−40.79	−43.90	−30.77	−9.63	−6.45	3.52	−30.76	−24.80	−13.57	−13.46	−11.91	4.86
VR	VR-056S	56,000	1,961,984.7	−47.69	−48.87	−37.49	−28.20	−12.23	19.42	−34.56	−35.65	−44.69	−49.73	−46.26	−60.78
VR	VR-050M	50,000	21,285,62.8	−33.15	−32.12	−15.63	−8.50	−4.93	−15.5	−26.09	−29.13	−33.21	−34.41	−25.97	−89.55
VR	VR-052M	52,000	2,223,666.9	−29.19	−28.80	−10.64	−14.13	−2.10	12.89	−17.83	−11.24	−23.68	1.73	6.15	26.7
VR	VR-053M	53,000	2,256,744.8	−11.80	−1.82	31.12	46.77	40.32	32.6	−28.09	−14.92	−19.07	−22.24	−17.36	−87.47
VTC	VTC-150S	150,000	3,736,842.1	−56.30	−50.69	−38.9	−45.18	−39.43	−42	−0.59	−2.67	−10.17	−39.67	−39.99	−95.88
VC	VC-150M	150,000	4,544,610.3	−13.44	−18.31	14.06	−30.48	−27.79	−9.46	−35.69	−27.96	−18.1	−47.01	−46.57	−55.66

## Appendix C

Physical quantities and abbreviations in the article

μ, COF	friction coefficient
*pv*	surface pressure multiplied by velocity
PAN	poly-acrylic-nitrile
T	test bench
H	highway
V	vehicle
C	city
VT	vehicle + trailer
R	testing ring (test track)
RS	testing ring (test track) + hill start
TH	test on test bench simulating highway conditions
VC	vehicle test in city
VTC	vehicle with trailer test in city
VR	vehicle test on test track
VRS	vehicle test on test track with hill start
VTRS	vehicle with trailer test on test track with hill start
t	the time the clutch pedal is released
T_c_	transmitted clutch torque
ω_r_	rotational velocity difference
z	number of friction surfaces
R_m_	friction radius
F_m_	clamp load
W	thermal load of the clutch
r_dyn_	dynamic rolling radius
i_1stgear_	first gear ratio
i_diff_	differential ratio
n_eng_	engine speed
T_engmax_	maximum engine torque
χ	engine type dependent torque factor
T_drag_	drag torque
S	smallest facing size category
M	middle facing size category
L	large facing size category
d_i_	inner diameter
d_w_	working diameter
σ	calculated sigma deviation
Lt	measuring length–surface roughness
